# Global Capacity for Emerging Infectious Disease Detection, 1996–2014

**DOI:** 10.3201/eid2210.151956

**Published:** 2016-10

**Authors:** Sheryl A. Kluberg, Sumiko R. Mekaru, David J. McIver, Lawrence C. Madoff, Adam W. Crawley, Mark S. Smolinski, John S. Brownstein

**Affiliations:** Boston Children’s Hospital, Boston, Massachusetts, USA (S.A. Kluberg, S.R. Mekaru, D.J. McIver, J.S. Brownstein);; ProMED-mail, Brookline, Massachusetts, USA (L.C. Madoff);; University of Massachusetts Medical School, Worcester, Massachusetts, USA (L.C. Madoff);; Massachusetts Department of Public Health, Boston (L.C. Madoff);; Skoll Global Threats Fund, San Francisco, California, USA (A.W. Crawley, M.S. Smolinski)

**Keywords:** disease outbreaks, surveillance, disease notification, public health, communication, emerging infectious disease, outbreak detection, global, capacity

## Abstract

The speed with which disease outbreaks are recognized is critical for establishing effective control efforts. We evaluate global improvements in the timeliness of outbreak discovery and communication during 2010–2014 as a follow-up to a 2010 report. For all outbreaks reported by the World Health Organization’s Disease Outbreak News, we estimate the number of days from first symptoms until outbreak discovery and until first public communication. We report median discovery and communication delays overall, by region, and by Human Development Index (HDI) quartile. We use Cox proportional hazards regression to assess changes in these 2 outcomes over time, along with Loess curves for visualization. Improvement since 1996 was greatest in the Eastern Mediterranean and Western Pacific regions and in countries in the middle HDI quartiles. However, little progress has occurred since 2010. Further improvements in surveillance will likely require additional international collaboration with a focus on regions of low or unstable HDI.

## Abstract 2

Timeliness of global outbreak discovery and public communication have gradually improved, but progress has slowed in recent years.

Today’s abundance of publicly available data from Internet-based sources has inspired ambitious disease surveillance efforts. Online news articles, Internet search terms, and user-generated content provide a wealth of information that can indicate disease occurrence, syndromes, and transmission patterns. Scientists can now collect and translate these data sources into useable surveillance platforms (*1*,*2*).

Progress in disease surveillance has been influenced by disease, region, and major geopolitical events. One study of geographic differences in outbreak reporting found that freer press and greater Internet usage correlate with reduced reporting lags (*3*). In terms of geopolitical events, highly publicized outbreaks with potential for international spread can put pressure on countries to enhance their surveillance systems. For example, the 2003 severe acute respiratory syndrome (SARS) epidemic highlighted the importance of rapid public communication and underscored the risk for global spread of epidemics (*4*–*6*), prompting countries around the world to improve their disease detection and communication tools (*7*,*8*). The revision of the World Health Organization (WHO) International Health Regulations (IHR), inspired by the SARS epidemic and implemented in June 2007, was designed to require member states to strengthen core surveillance and response activities to protect against the international spread of disease (*9*); however, recent reports have documented widespread lapses in compliance with these regulations (*10*,*11*).

There are countless ways to measure change in disease surveillance capacity. A recent review of outbreak investigations across Europe explored timing from outbreak declaration to conclusion of the investigation (*12*). In 2010, Chen et al. published a report estimating time from outbreak start to discovery and public communication across the world (*13*). This updated report applies similar methods. No single metric can provide a definitive account of surveillance improvements, but combined, they depict a more comprehensive picture.

The report by Chen et al. found a significant improvement in timeliness of surveillance for outbreaks around the world during 1996–2009 (*13*). Median times from outbreak start to discovery and public communication were 23 and 32 days, respectively. The time from the start of an epidemic until its discovery improved by an average of 7.3% per year, and the time to public communication about the epidemic improved by 6.2% per year (*13*).

Since that time, the world has seen several large outbreaks (e.g., cholera in Haiti and Ebola in West Africa), several emergences of new pathogens (e.g., Middle East respiratory syndrome and avian influenza H7N9), and some reemergence of established pathogens (e.g., poliomyelitis in the Middle East and Africa). The past few years have also seen further development of digital surveillance tools, with increased data volume on social media sites (e.g., Twitter and Facebook), more timely updating of news sources, and increased citizen science reporting.

Our objective was to evaluate the trends in disease surveillance in recent years, building on methods established in the earlier work, to assess whether timeliness of outbreak detection and communication has continued to improve. We examined whether more recent changes have been global in scope or confined to certain regions or whether they are aligned with certain factors, such as Human Development Index (HDI) quartile.

## Methods

### Data

We replicated the data collection methods described in Chan et al. (*13*). Outbreaks of interest were those listed in the WHO’s Disease Outbreak News (*14*) from January 1996 through December 2014 that fit our selection criteria. Based on predetermined exclusion criteria, we removed outbreaks of ongoing, endemic, or seasonal diseases; isolated or single cases; diseases occurring only in animals; foodborne outbreaks; nonnatural cases (e.g., acts of bioterrorism and laboratory accidents); and noninfectious health events (*13*). 

For each outbreak meeting our selection criteria, we identified corresponding reports from 3 informal disease reporting systems and abstracted data about the type of outbreak and relevant milestones from outbreak emergence to discovery, laboratory confirmation, communication, and WHO verification. These 3 sources were the Program for Monitoring Emerging Diseases (ProMED), the Global Public Health Intelligence Network (GPHIN), and HealthMap. ProMED is an expert-moderated global electronic reporting system that collects information about disease outbreaks and acute toxin exposures from local media, local and regional observers, online sources, and official reports (*15*). GPHIN is an early warning network operated by the Public Health Agency of Canada that retrieves and categorizes online news articles about any health hazards across 9 languages (*16*). HealthMap is an Internet-based, largely automated disease surveillance system that collects infectious disease information from various official and informal electronic sources across 15 languages and categorizes them by disease and geography (*17*).

Additionally, we searched Google, Google Scholar, and PubMed to fill in missing outbreak start and discovery milestones, which was not done in the 2010 study. We did not expand our search for date of public communication because we were primarily interested in communications identified by the 3 informal disease reporting systems.

As proxies for local contextual factors, such as government transparency and health system infrastructure, we collected country-level HDI scores from the United Nations Development Program website for all available years during 1990–2013 (*18*) and annual polity data from the Center for Systemic Peace Polity Project website (*19*). Although these are imperfect substitutes, we are not aware of any variables that capture all contextual nuances at a useful geographic and temporal granularity. The HDI combines life expectancy, years of schooling, and gross national income per capita into a summary measure of achievement in human development. We compared the rank of each country in 1990 and 2013 to create a rank change value that we used to construct quartiles, with quartile 1 representing the greatest rank improvement and quartile 4 representing the greatest rank decline. The polity scale provides a measure of democratic authority among governing authorities, ranging from −10 for a hereditary monarchy to 10 for a fully institutionalized democracy (*19*). Studies have found associations between HDI and health system quality, health outcomes, disease prevalence, and health-seeking behavior (*20*–*24*). Polity has been associated with health metrics such as healthcare expenditures and infant mortality (*25*,*26*).

### Covariates

Primary milestones of interest were the dates of outbreak start, outbreak discovery, and public communication about the outbreak. A list of outbreak events was used to estimate each of these milestone dates (Table 1). Definitions of all dates and milestones were consistent with those used by Chan et al. (*13*).

**Table 1:** Milestones of interest and events used to estimate their dates in a study assessing global capacity for emerging infectious disease detection, 1996–2014

Milestone	Defined as earliest of
Date of outbreak start	• Symptom onset • Hospitalization or medical visit
Date of outbreak discovery	• WHO report • ProMED source • HealthMap source • GPHIN source • Announcement by a local authority figure or medical professional • WHO notification • Hospitalization or medical visit • Laboratory confirmation • Preliminary laboratory confirmation • Declaration of an epidemic • Alert raised • Earlier mentioned announcement date
Date of public communication	• WHO report • ProMED source • HealthMap source • GPHIN source • Announcement by a local authority figure or medical professional • Declaration of an epidemic • Alert raised • Earlier mentioned announcement date

*GPHIN, Global Public Health Intelligence Network; ProMED, Program for Monitoring Emerging Diseases; WHO, World Health Organization.

### Statistical Analysis

#### Timeline of Outbreak Progression

To characterize the timeline of outbreak progression, we calculated the median time from outbreak start date to each of 4 milestones: 1) outbreak discovery (i.e., discovery delay); 2) public communication about the outbreak (i.e., communication delay); 3) laboratory confirmation; and 4) Disease Outbreak News report about the outbreak (as a proxy for WHO verification). The 95% CIs for the median values were determined by using the bootstrapping method with 1,000 replicates.

#### Geographic, Temporal, Development, and Polity Trends

For the outcomes of discovery and communication delay, we calculated median values (with bootstrapped 95% CI) for each year during 1996–2014 and for the categorized periods before and after the WHO’s revised IHR went into effect on June 15, 2007. We also explored heterogeneity by WHO geographic region (*27*), quartile of change in HDI across the study period, and quartile of polity score (based on country and year of the outbreak) (*19*). Region-, HDI-, and polity-stratified values were plotted as Loess curves over time with a smoothing parameter automatically selected to balance residual sum of squares with the complexity of the fit (*28*).

We assessed rates of change in time to each milestone by using univariable Cox proportional hazards regression analysis with discovery delay and communication delay as outcomes for 2 separate models and outbreak start date as the predictor variable for both models. This model produced a daily hazard of change in surveillance timeliness from 1 date to the next, and we multiplied the result by 365 and exponentiated it to calculate an annual hazard ratio (HR) of change in timeliness from year to year. Outbreaks with missing dates of discovery or public communication were excluded. We repeated the analysis stratified by WHO region, change in HDI quartile, and country-specific polity quartile in the year of the outbreak.

#### Sensitivity Analysis

We assessed the validity of our HRs by the same method described in Chan et al. (*13*). We ran Cox proportional hazards regression models comparing time to our milestones before and after June 15 of each year, the date on which the revised IHR were implemented in 2007.

## Results

Of 109 WHO Disease Outbreak News reports from 2010 through 2014 not titled as updates, we identified 73 (67%) that fit our inclusion criteria, of which 66 (61%) had reportable start dates. The geographic distribution of outbreaks was similar to that of the 281 outbreaks from 1996 through 2009. For all 347 outbreaks (Table 2) combined that fit the selection criteria and had known start dates, 54% were from Africa, 11% from the Western Pacific, 10% from the Eastern Mediterranean, 10% from the Americas, 7% from Europe, and 7% from South-East Asia. Change in HDI rank was stratified as −58 to −34; −33 to −14; −13 to −3; and −2 to +22. Polity score quartiles were stratified as −10 to −3; −2 to 0; 1 to 7; and 8 to 10. HDI change values were missing for 21% of outbreaks, and polity scores were missing for 11% of outbreaks.

**Table 2:** Type and number of disease outbreaks meeting the selection criteria in a study assessing global capacity for emerging infectious disease detection, 1996–2014

Disease	No. outbreaks, N = 347
Anthrax	1
Avian influenza (H9N2)	1
Chikungunya	4
Cholera	93
Dengue	13
Diphtheria	1
Dysentery	1
Enterovirus D68	1
H5 influenza	39
H7 influenza	3
Hand, foot, and mouth disease	4
Hemorrhagic fevers (Crimean-Congo, Ebola, Marburg, undiagnosed)	29
Hantavirus	2
Henipavirus	4
Hepatitis E	2
Japanese encephalitis	3
Lassa fever	2
Legionellosis	4
Leptospirosis	3
Louseborne typhus	1
Lujo virus	1
MERS	2
Malaria	3
Measles	3
Meningitis	24
Monkeypox	1
Nonavian influenza A	4
O'nyong-Nyong fever	1
Plague	10
Poliomyelitis	24
Relapsing fever	1
Rift Valley fever	6
SARS	3
Shigellosis	4
*Streptococcus suis*	1
Tularemia	2
Typhoid fever	4
West Nile virus	3
Yellow fever	39

*MERS, Middle East respiratory syndrome; SARS, severe acute respiratory syndrome.

Our primary outcomes of discovery delay and communication delay for all outbreaks from 1996 through 2014 had median times of 20 days (95% CI 16–25 days) and 32 days (95% CI 29–38 days), respectively. Median time to laboratory confirmation was 36 days (95% CI 32–46 days) and to WHO verification was 49 days (95% CI 44–55 days). These outcomes varied by WHO region, change in HDI quartile, and country-specific polity quartile in the year of the outbreak, and across time. For both primary milestones, median times were longest for Africa and the Eastern Mediterranean and shortest for the Western Pacific and South-East Asia (Table 3). Median discovery delay decreased monotonically with improving HDI rank quartile. Communication delay was uniform across the HDI variable aside from a large increase for the quartile with the greatest decline in HDI rank (Table 4). Polity showed a less clear trend; time lags increased with decreasing polity from the first through third polity quartiles, and then decreased for the quartile of lowest polity (Table 5).

**Table 3:** Median days to disease discovery and public communication, by region, in a study assessing global capacity for emerging infectious disease detection, 1996–2014

Region	No. outbreaks*	Median no. days to discovery (95% CI)	No. outbreaks*	Median no. days to communication (95% CI)
All†	342	20 (16–25)	346	32 (29–38)
Africa	175	27 (20–31.5)	177	43 (32–51)
Americas	31	18 (12–29)	31	23 (18–33)
Eastern Mediterranean	39	26 (6–41)	39	39 (18–56.5)
Europe	25	20 (7–33)	25	31 (18–77)
South East Asia	24	13 (5–30)	25	15 (11–36)
Western Pacific	47	5 (4–7.3)	48	19 (12.5–31.5)

*Includes all outbreaks with known start date. Those without a known discovery date are excluded from the calculation of days to discovery.  †Includes colonies/territories/countries without a World Health Organization region designation.

**Table 4:** Median days to discovery and public communication, by quartile of change in rank in the Human Development Index, in a study assessing global capacity for emerging infectious disease detection, 1996–2014

HDI rank change quartile	No. outbreaks†‡	Median no. days to discovery (95% CI)	No. outbreaks*	Median no. days to communication (95% CI)
Q1: Most improvement	49	5 (4–14.5)	49	20 (13–33)
Q2: High-intermediate	43	11.5 (6.8–19)	44	21.5 (15–28)
Q3: Low-intermediate	60	21 (14–33)	61	23 (15–40.5)
Q4: Most decline	120	26 (17–32)	121	48 (32–58)

*HDI, Human Development Index. †Quartiles are defined based on all countries with HDI scores from 1990 and 2013, regardless of whether they had outbreaks. Therefore, outbreaks are not evenly distributed across the quartiles. ‡Outbreaks without a known discovery date are excluded from the calculation of days to discovery.

**Table 5:** Median days to discovery and public communication, by quartile of polity, in a study assessing global capacity for emerging infectious disease detection, 1996–2014

Polity quartile	No. outbreaks*	Median no. days to discovery (95% CI)	No. outbreaks*	Median no. days to communication (95% CI)
Q1: Highest polity	67	17 (10–23.3)	67	23 (17–30)
Q2: High-intermediate	84	23.5 (15–32)	87	32 (26–48)
Q3: Low-intermediate	67	35 (22.3–51)	67	47 (33–64)
Q4: Lowest polity	87	10 (4–22)	88	32.5 (22.3–44.5)

*Quartiles are uneven because polity score is ordinal, not continuous.

**Figure 1:**
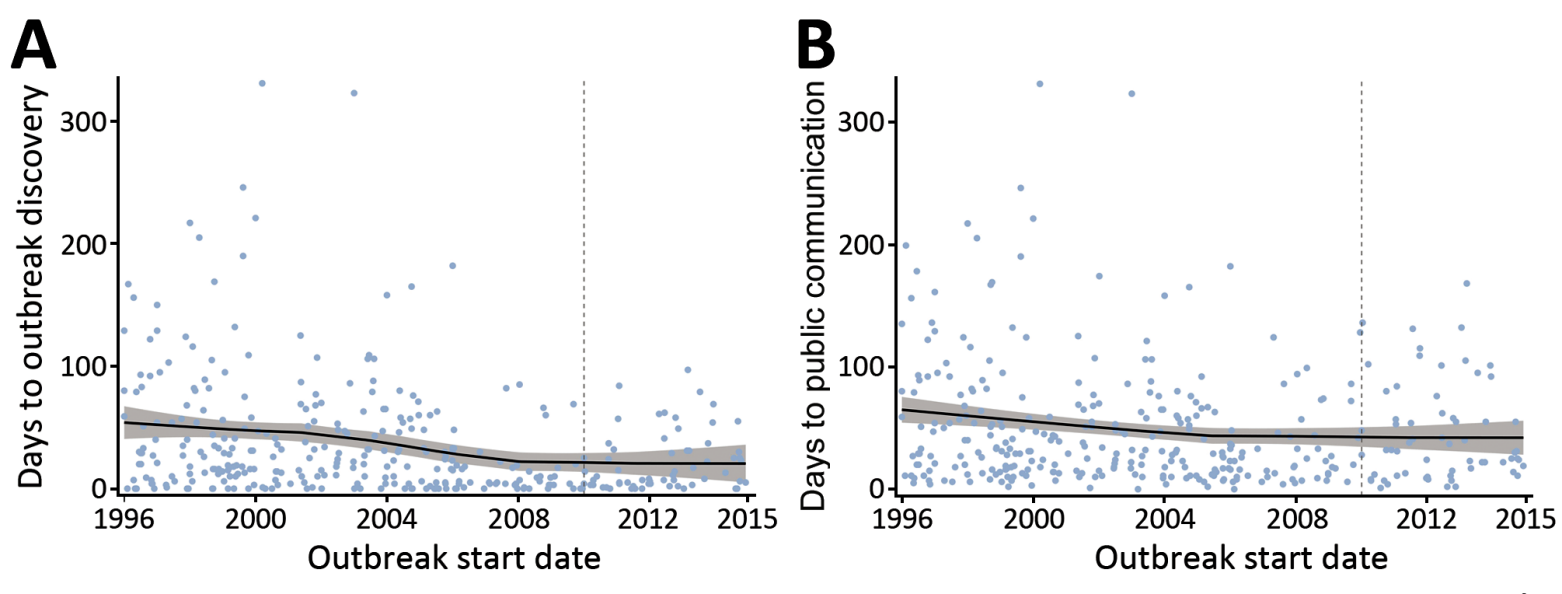
Scatterplots with Loess curves of time to A) outbreak discovery and B) public communication in a study assessing global capacity for emerging infectious disease detection, 1996–2014. Gray shading around curve indicates 95% CI. Dashed line marks the beginning of the 5-year period of this study.

**Figure 2:**
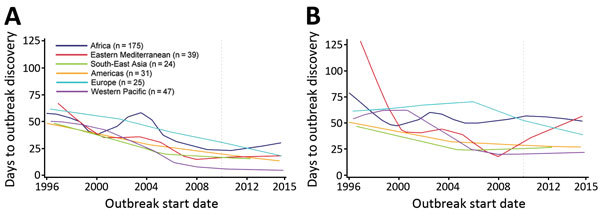
Loess curves of time to A) outbreak discovery and B) public communication, by World Health Organization region, in a study assessing global capacity for emerging infectious disease detection, 1996–2014. Dashed line marks the beginning of the 5-year period of this study.

**Figure 3:**
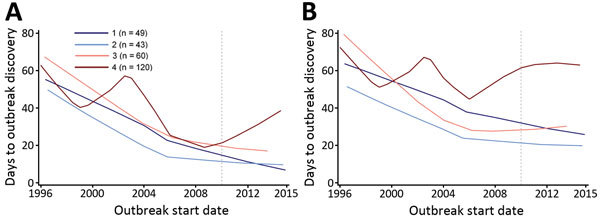
Loess curves of time to A) outbreak discovery and B) public communication, by quartile of change in Human Development Index rank, in a study assessing global capacity for emerging infectious disease detection, 1996–2014. Dashed line marks the beginning of the 5-year period of this study.

Discovery and communication delays generally decreased over time but exhibited large fluctuations and substantial uncertainty because of the small number of outbreaks (Figure 1). Descriptive regional Loess curves show an overall trend of shortened discovery delay for each region but no clear trend for communication delay (Figure 2). However, the occurrence of extreme lags, defined as outliers relative to all recorded lags (>102-day and >117-day discovery and communication delays, respectively), has declined over time; the average annual number of extreme discovery delays decreased from 2.5 to 0 after 2007, and the average annual number of extreme communication delays decreased from 2.4 to 0.75. Our HDI-quartile Loess curves illustrate that progress in surveillance systems might vary according to changes in country-level human development metrics (Figure 3).

Cox regression estimates show that discovery and communication delays significantly decreased over time, with an average annual reduction of 5.5% in discovery delay (HR 1.055, 95% CI 1.034–1.076) and 2.5% in communication delay (HR 1.025, 95% CI 1.006–1.045). Days to laboratory confirmation, available for 82% of outbreaks, decreased by an average of 3.6% per year (HR 1.036, 95% CI 1.014−1.060), and days to WHO verification decreased by an average of 2.1% per year (HR 1.021, 95% CI 1.001–1.041).

The Eastern Mediterranean and Western Pacific regions showed the greatest improvements in discovery and communication delays, whereas Europe showed the least improvement in discovery delay, and Africa, Europe, South-East Asia, and the Americas all showed little improvement in communication delay (Table 6). When evaluated according to quartile of change in HDI rank, the 2 middle HDI quartiles exhibited the greatest improvement for both indicators (Table 7).

**Table 6:** Results of univariate Cox proportional hazards regression analyses, overall and by region, in a study assessing global capacity for emerging infectious disease detection, 1996–2014

Region	No. outbreaks	Median no. days to discovery (95% CI)	No. outbreaks	Days to communication hazard ratio (95% CI)
All*	342	1.06 (1.03–1.08)†	346	1.03 (1.01–1.05)†
Africa	175	1.05 (1.02–1.08)†	177	1.01 (0.98–1.04)
Americas	31	1.06 (0.99–1.13)	31	1.03 (0.97–1.10)
Eastern Mediterranean	39	1.08 (1.01–1.15)†	39	1.06 (1.00–1.13)†
Europe	25	1.04 (0.96–1.12)	25	1.02 (0.95–1.10)
South-East Asia	24	1.06 (0.97–1.15)	25	1.03 (0.94–1.11)
Western Pacific	47	1.07 (1.01–1.14)†	48	1.08 (1.02–1.14)†

*Includes colonies/territories/countries without a WHO region classification. †Statistically significant (α = 0.05).

**Table 7:** Results of univariate Cox proportional hazards regression analyses, by quartile of change in Human Development Index (HDI) rank, in a study assessing global capacity for emerging infectious disease detection, 1996–2014

HDI rank change quartile	No. outbreaks*	Days to discovery hazard ratio (95% CI)	No. outbreaks*	Days to communication hazard ratio (95% CI)
Q1: Most improvement	49	1.04 (0.98–1.09)	49	1.04 (0.98–1.09)
Q2: High-intermediate	43	1.09 (1.03–1.15)†	44	1.06 (1.00–1.12)†
Q3: Low-intermediate	60	1.08 (1.03–1.13)†	61	1.07 (1.02–1.12)†
Q4: Most decline	120	1.05 (1.01–1.08)†	121	1.00 (0.97–1.03)

*Quartiles are defined based on all countries with HDI scores from 1990 and 2013, regardless of whether they had outbreaks. Therefore, outbreaks are not evenly distributed across the quartiles. †Statistically significant (α = 0.05).

A Cox regression model with an indicator for whether the outbreak occurred before or after June 2007, when the WHO’s revised IHR were implemented, shows an 84% reduction in discovery delay after IHR implementation (HR 1.84, 95% CI 1.44–2.35) but no significant change in communication delay (HR 1.18, 95% CI 0.93–1.49). However, our sensitivity analysis comparing the reduction in discovery delay before and after June of other years shows that improvement peaked in 2005, 2 years before the IHR were implemented (HR 2.01, 95% CI 1.60–2.53).

## Discussion

Our results confirm that improved timeliness of outbreak discovery found by Chen et al. (*13*) has generally been sustained, but with smaller improvements since 2010. Median discovery delay and communication delay fluctuated over time; however, after we stratified by WHO region and HDI rank change, clear downward trends appear for most strata. We have also seen fewer extreme delays in recent years. Furthermore, all regional Cox regression models showed increases in timeliness. Although some were not statistically significant, partly because of the small numbers of outbreaks, we visualize the downward trend with Loess curves.

Because surveillance capabilities vary by region, yearly regional distribution of outbreaks has a strong influence on aggregate global outcomes. Although the majority of outbreaks in most years originated in Africa, where outbreak discovery tends to be slower (Table 3), there were years in which another region also had a large number of outbreaks, albeit fewer than Africa (e.g., Eastern Mediterranean in 2006 and Western Pacific in 2008), and years in which another region experienced more outbreaks than Africa (e.g., Western Pacific in 2007).

Despite regional disparities in timeliness of disease discovery, all regions showed some improvement. Variability between regions probably resulted from a combination of differences in culture, Internet availability, and previous outbreak experiences. Public communication must follow disease discovery, and additionally depends on recognition of outbreak severity, willingness to expose the outbreak, and belief that outbreak control will improve after the announcement. The 1996 start date of our study was during the early days of Internet-based surveillance, but some countries such as the United States, France, England, Norway, Scotland, and Sweden were already using digital technologies for centralizing and aggregating data (*29*–*32*), which set them ahead of the surveillance curve but left them with less room for improvement over the 18-year study period. We expect that local phenomena, such as the proliferation of Internet usage or the occurrence of particularly alarming regional outbreaks, were associated with local surveillance improvements, whereas global phenomena, such as the SARS epidemic and the adoption of the IHR (2005), encouraged more widespread surveillance system development.

Our analyses stratified by HDI and polity shed light on the role of government characteristics in surveillance system strength. Change in HDI rank from 1990 to 2013 captures a sustained movement toward improved (or stymied) development. Polity in the year of the outbreak, however, illustrates a time-constrained snapshot of government status. Although our analysis across levels of polity did not reveal noteworthy trends (Table 8), we found that the direction of change in HDI over time had a strong relationship with surveillance system success. Median discovery delay increased with worsening HDI quartile. Outbreaks in countries with the most positive change in HDI rank had the shortest median time to detection but the least reduction in delay (consistent with having the least room for improvement). The middle quartiles improved significantly, whereas the countries with the greatest decrease in HDI, or the most political and social instability, showed less improvement. Meanwhile, median communication delay was uniform across the HDI variable, aside from the lowest quartile, which had a significantly longer delay. That quartile was also the only quartile to show no improvement over time, although this was not significant (Figure 3, panel B). Although these results suggest that long-term national systemic improvements might foster surveillance system progress, it might be practical to begin with increased availability of and education regarding moderated digital disease reporting platforms. Additionally, international agencies can encourage surveillance efforts by bolstering consensus regarding transparency and developing improved mechanisms to enforce adherence to existing regulations.

**Table 8:** Results of univariate Cox proportional hazards regression analyses by quartile of polity, 1996–2014

Polity quartile	No. outbreaks*	Days to discovery hazard ratio (95% CI)	No. outbreaks*	Days to communication hazard ratio (95% CI)
Q1: Highest polity	67	1.04 (0.99–1.10)	67	1.01 (0.96–1.06)
Q2: High-intermediate	84	1.04 (1.00–1.09)	87	1.00 (0.96–1.05)
Q3: Low-intermediate	67	1.06 (1.00–1.13)†	67	1.05 (0.99–1.11)
Q4: Lowest polity	87	1.08 (1.04–1.13)†	88	1.03 (1.00–1.07)

*Quartiles are uneven because polity score is ordinal, not continuous. †Statistically significant (α = 0.05).

Our finding of sustained improvement in timeliness of outbreak discovery for all regions in the current IHR period is encouraging. We can see that improvements in the lead up to adoption of the revised IHR have been maintained with some year-specific fluctuation, and extreme delays have been eliminated. There are likely several drivers behind these improvements, including enhancement of local surveillance systems to meet the requirements of the revised IHR, and continued development of informal surveillance sources such as GPHIN, ProMED, and HealthMap (*33*), and almost universal increases in human development scores.

We found that global and stratified regional trends were attenuated relative to our findings from 5 years ago, suggesting that progress has slowed. This finding is expected, given that there is now less room for improvement; however, different strategies will now be required to achieve further success. Initial drops in discovery and communication delays might have been partly attributable to the availability of digital surveillance technology, but further progress will require developments in local surveillance infrastructure. These include bolstering leadership and coordination, increasing access to medical care, and building trust in health systems, all costly and complex endeavors (*34*). Increased international collaboration can help diffuse the effort required for these undertakings (*35*).

We should note that the relatively small improvements in time to laboratory confirmation and WHO verification are not unexpected. Speed of laboratory testing varies considerably by disease and geography because of availability of laboratory capacity, particularly in the case of an unusual or nonendemic disease. Furthermore, because WHO verification generally requires laboratory confirmation and consent of national authorities, this announcement might also be delayed. Given that 33% of first public communications were WHO reports, it is not surprising that the timeliness of WHO report closely reflects the trend in timeliness of public communication.

Despite the general progress in timeliness of disease discovery, we find that there is room for improvement in timely public communication about outbreaks. Although the aggregate Cox regression shows an average improvement of 2.4% per year, we see that Africa and Europe barely improved at all, whereas South-East Asia and the Eastern Mediterranean improved initially but seem to have slowed in later years. Although detection of an outbreak triggers the initial alert for the surveillance system, it is public communication that will elicit a local response among the susceptible population and an international response among those governments and agencies that are equipped to assist in outbreak containment. In 2003, the first reports of SARS occurred >2 months after the first cases were discovered, after >300 people in China were already infected (*36*). In Saudi Arabia, Middle East respiratory syndrome was not announced until ≈3 months after the first symptomatic patient sought medical attention because of a lack of a definitive laboratory diagnosis (*37*). Similarly, the first cases of Ebola in Guinea in 2014 were not initially diagnosed, causing a delay of several months before the government recognized the outbreak of hemorrhagic fever and published an announcement (*38*). This delay provided time for the disease to spread to the large capital city of Conakry before control measures could be taken, at which point it became an unprecedented challenge to combat the outbreak (*39*).

The results of our analysis are subject to a few limitations. The small annual numbers of eligible outbreaks prevented us from stratifying our analyses by factors that might be associated with surveillance capacity, such as disease type (e.g., respiratory and hemorrhagic) or country-specific statistics on Internet usage. Additionally, many confounders or modifiers of interest, such as outbreak severity and size, local concurrent disease burden, and health system strength, are not available as validated metrics for analysis. Regions with the least room for improvement would have benefited by further stratification as a means to identify focus areas. We hope that future research will shed light on some of the more intricate heterogeneities in surveillance success around the world. The analysis was also limited by a large number of unreported outbreak start and discovery dates. We followed estimation procedures used by Chan et al. (*13*) to maximize the number of outbreaks we could include for our analysis, recognizing that our estimated dates were not entirely accurate. This approximation likely led to conservative estimates (i.e., overestimated times to surveillance milestones), with a stable degree of misclassification over time, thus introducing minimal bias on our final hazard ratios.

Our findings illustrate the general improvement in timeliness of outbreak discovery and the need for further improvement in timeliness of public communication. However, it is important that our conclusions be understood in the context of disease- and region-level heterogeneity. Although our data describe a single measure of progress in disease surveillance, we hope that our findings are considered in light of other research on contextually appropriate measures and indicators. We highlight the importance of international efforts to enforce regulations, identify regional strengths and weaknesses, and set appropriate goals for surveillance system strengthening, so that when an outbreak does occur, control measures can be set in motion quickly enough to avoid local and potentially pandemic disease spread.
